# Definition of Impulsivity and Related Terms Following Traumatic Brain Injury: A Review of the Different Concepts and Measures Used to Assess Impulsivity, Disinhibition and other Related Concepts

**DOI:** 10.3390/bs4040352

**Published:** 2014-10-09

**Authors:** Andrea Kocka, Jean Gagnon

**Affiliations:** 1 Department of Psychology, University of Montreal, Montreal, QC H3C 3J7, Canada; E-Mail: jean.gagnon@umontreal.ca; 2 Centre for Interdisciplinary Research in Rehabilitation of Greater Montreal (CRIR), Montreal, QC H2H 2N8, Canada; 3 Centre de recherche en neuropsychologie et cognition (CERNEC), Montreal, QC H3C 3J7, Canada

**Keywords:** impulsivity, disinhibition, TBI, brain injury, impulse control, regulation, inhibitory control, definitions, UPPS

## Abstract

Impulsivity is a common and debilitating sequela following traumatic brain injury (TBI), but there is no consensual definition or measure to assess this construct. The following review aims to elucidate the differences and resemblances between impulsivity, disinhibition and other related terms following brain injury and the instruments that are commonly used to measure these constructs. To do so, a search through different databases was conducted in order to find articles that mention and define impulsivity, disinhibition, impulse control, regulation deficits, dyscontrol and risky behavior. The concepts that stand out from the literature, the measures used, the similarities, the differences between these concepts are observed. The fit with the UPPS model of impulsivity, according to which impulsivity is a multidimensional concept composed of four distinct dimensions (urgency, perseverance, premeditation and sensation-seeking) is discussed.

## 1. Introduction

Impulsivity is a common consequence following traumatic brain injury (TBI) and has many repercussions on the patient’s and on their relative’s quality of life [1], on the patient’s social and professional outcomes [2], on the patient’s safety [3], on the rehabilitation process [4] and on the cost of healthcare [5]. It is therefore particularly important to properly identify the presence of impulsivity in post-TBI patients.

However, despite the clinical importance of an accurate evaluation, there is no valid instrument designed to specifically measure impulsivity following TBI [6]. This can, at least in part, be explained by the absence of a consensual definition of impulsivity. Firstly, as Whiteside and Lynam put it [7], the term “impulsivity” suffers with the “jingle” and “jangle” fallacies [8] which means that the same label can refer to different concepts and that two different labels can in fact refer to the same concept. Secondly, as Rochat *et al.* pointed out, behaviors considered to be impulsive are heterogeneous [6]. Thirdly, a specific behavior may have different aetiologies, not all of which are impulsivity related. For example, aggressive behavior might be impulsive or episodic, the second being related to epileptogenesis [9]. Similarly, some definitions of impulsivity that are closely linked to cognitive mechanisms (*i.e.*, executive functions) and measured with performance tasks are not always correlated to actual observable impulsive behaviors.

Moreover, in the TBI literature specifically, different terms are used to describe phenomena that may or may not resemble impulsivity depending on the author’s definition of impulsivity. In that regard, some studies address impulsivity in the terms of personality change while others address it in terms of executive dysfunction. Disinhibition and impulsivity are sometimes used interchangeably and other times used to illustrate separate concepts. Behavioral and emotional changes, dyscontrol, lack of impulse control are only a few other examples. Also, depending on the definition of impulsivity and the dimensions one aims to assess, certain measures might be more relevant than others in order to identify impulsivity [10]. It should also be noted that different dimensions of impulsivity have been linked to different types of detrimental outcomes. For example, motor impulsivity is likely to have an impact on physical well-being and verbal impulsivity on interpersonal relationships [10].

Finally, the idea that impulsivity is a multidimensional concept, such as the UPPS model which is a four dimensional (urgency, perseverance, premeditation and sensation-seeking) model of impulsivity [7], is relatively new in the TBI literature and has not been thoroughly studied as of yet.

The following article aims to elucidate the differences and resemblances between impulsivity, disinhibition and other related terms following brain injury and the instruments that are commonly used to measure these constructs.

## 2. Method

An initial literature review was conducted in order to identify all the terms that seemed relevant to impulsivity following TBI. The words that were selected are the following: Impulsivity, impulsiveness, emotion regulation, behaviour problems, behavior problems, behaviour sequalae, behavior sequalae, dyscontrol, impulse control, disinhibition and impulse control. Each of these words was combined (AND) with TBI, brain injury, acquired brain injury, traumatic brain injury, ABI and brain trauma. These combinations were then run through different databases (Ovid, EBSCO and ISI) and all the publications that seemed pertinent were selected until a point of saturation was reached. A total of 4783 results were generated out of which 347 were kept based on relevance after a pre-screening (title and keywords).

In an attempt to have the most inclusive literature possible, we also searched for articles containing keywords that could, more broadly, be linked to impulsivity after TBI. Those keywords are Dysexecutive, Inhibition and Personality. Once again, each of those keywords was combined (AND) with the following: TBI, brain injury, acquired brain injury, traumatic brain injury, ABI and brain trauma. This step generated an additional 5503 results, out of which 222 were kept.

Finally, we also searched through the references, the citing and the related articles of those found previously. Once again, a point of saturation was reached.

After a thorough screening based on abstract information during which only articles that seemed pertinent to the population we were interested in (*i.e.*, at least some adult TBI patients in the sample) and the elimination of duplicates, 124 articles were kept for the present review. Each of these 124 articles was then analyzed to see if the authors used and/or defined the constructs below.

## 3. Definitions and Measures

### 3.1. Definitions

#### 3.1.1. Impulsivity/Impulsiveness

Even though impulsivity is broadly understood as a concept that encompasses a multitude of behaviors or responses that are poorly conceived, premature, inappropriate, and that frequently result in unwanted or deleterious outcomes [11], in the articles selected for this current review, most authors refer to impulsivity as a multidimensional construct. This seems to be a general consensus in the current literature [12,13]. However, the dimensions of interest vary considerably from one study to the next. Here is a brief description of the different studies and their respective definition of impulsivity.

Firstly, Barratt’s Impulsiveness Scale (BIS) derived from Barratt’s three factor model of impulsivity [14,15] is a self-report questionnaire and seems to be the most commonly used to assess impulsivity in a TBI population. According to this model, there are three dimensions to impulsivity: Motor impulsivity which refers to acting without thinking, cognitive impulsivity which refers to quick decision taking and non-planning impulsivity which refers to a present orientation. McHugh and Wood examined the relation between impulsivity and decision making after brain injury using the BIS-11 [16]. They demonstrated that the TBI group’s decision-making was more impulsive than the control group’s and that the TBI participants scored higher on all three dimensions of the BIS-11. Greve and collaborators also used the BIS-11 this time to differentiate TBI patients at risk for impulsive aggression from those who are not [4] and in another study aiming to observe the use of cognitive strategies in TBI patients with problems with impulsive aggression [17]. Similarly, Floden, Alexander, Kubu, Katz, & Stuss used the BIS-11 in an effort to distinguish impulsivity from risk-taking [18]. They, however, specified that their definition of impulsivity is closer to the motor and non-planning subscales. Ferguson and Coccaro, in a study aiming to determine if a history of mild or moderate TBI was associated with impulsivity and aggression, also used the BIS-11 [19].

In another study of impulsivity, Votruba *et al.* aimed to assess impulsivity after TBI using different measures (*i.e.*, rating scales, questionnaires and performance tasks) in relation to direct behavioral observation [10]. To do so, they focused on the mode of expression of these impulsive behaviors, either motor or verbal. They define an impulsive act (motor impulsivity) as being an action that the patient performs spontaneously, without evidence of preconsideration, and that has potential for negative consequences for the patient or others. More specifically, the authors observed dangerous acts, impersistences of action, disruptive behaviors, inappropriate acts, sexual actions, self-injurious actions and perseverated actions. According to the same authors, an impulsive statement (verbal impulsivity) is a statement made spontaneously, without evidence of preconsideration, with potential for negative consequences for the patient or others. Votruba and colleagues specifically targeted impersistent statements, inappropriate interruptions, inappropriate statements, sexual statements and perseverated statements for this dimension. The results of this study suggest that, even though rating scales completed by rehabilitation therapists (as opposed to self-reports) converged with verbal impulsivity and some performance tasks converged with motor impulsivity, direct observation of behaviors is the most accurate measure of impulsivity.

Similarly, in a study by Aeschleman and Imes, impulsivity was assessed by direct observation using four distinct categories [20]. These categories were verbal impulsivity which was defined as yelling out abusive comments or verbally threatening to engage in destructive behavior, gestural impulsivity which required the use of body language to convey threat or insult, physical impulsivity which was operationalized as striking out at another person or object and making actual physical contact with person/object, tempting to strike out but missing the target, or throwing objects and finally a category named “other” which, according to the authors, includes other incidents in which the participant clearly acted in an impulsive manner, but the behavior does not fall into the above categories (e.g., walking out of class). The first two categories are similar to those defined in the study conducted by Votruba and colleagues [10].

Rochat and colleagues, as for them, use a very different definition of impulsivity [6]. In their studies on post-TBI impulsivity, they refer to Whiteside and Lynam’s conceptualization of impulsivity [6,13]. According to this specific theory, which is based on the Five Factor Model of personality [21], there are four dimensions to impulsivity which can be measured with a rating scale (either self-report or relative-report) called the UPPS scale. Urgency refers to the tendency to experience and act on strong impulses frequently under conditions of negative affect [22] and positive affect [23], (lack of) premeditation refers to the inability to think and reflect on the consequences of an act before engaging in that act [22], (lack of) perseverance refers to an individual’s inability to remain focused on a task that may be boring or difficult [22] and finally sensation seeking refers to the tendency to enjoy activities that are exciting and to the willingness to try new experiences. Rochat and colleagues have not only demonstrated that the relative-reported version of this questionnaire had adequate factor structure in a TBI sample, but have also shown, as expected, that urgency, (lack of) premeditation and (lack of) perseverance increased following brain injury and that sensation seeking decreased [6].

The Dysexecutive questionnaire (DEX) [24] is a measure of executive dysfunction frequently used in TBI studies [25,26,27,28] In this questionnaire, impulsivity is assessed by a single question and is defined as acting without thinking and doing the first thing that comes to mind.

In a study in which the nursing staff observed adverse behaviors in a rehabilitation setting in patients who have suffered from TBI or stroke [29], a behavior rating form developed specifically for the present study was used. Adverse behaviors such as restlessness, wandering, impulsiveness and verbal aggression where observed. Impulsiveness was defined as sudden movements or motions that indicated lack of behavioral or verbal control over oneself and was among the most observed and frequent adverse behaviors.

In the Behavioral Dysexecutive Syndrome Inventory, a structured interview, impulsivity, combined with irritability and aggressiveness, is considered to be one of 12 domains of interest in the evaluation of behavioral changes by informants of patients who have suffered from severe traumatic brain injury, stroke, mild cognitive impairment, Alzheimer disease, multiple sclerosis, and Parkinson disease [30]. The authors do not, however, present a definition of impulsivity in this study.

Dixon and colleagues [31,32,33] use a very different narrow definition of impulsivity. In their studies of patients who have suffered from TBI, impulsivity is conceptualized as the selection of a smaller reinforcer that comes in a shorter delay rather than a delayed larger reinforcer.

#### 3.1.2. Impulse Control

In their clinical model of executive functions, Sohlberg and Mateer classify impulse control as part of response inhibition [34]. According to these authors, response inhibition is the ability to inhibit automatic or prepotent response tendencies and is critical for flexible goal-directed behavior. An impairment in response inhibition may result in impulsive responding, stimulus-boundedness and perseveration.

It is interesting to compare this definition to the psychiatric comprehension of impulse control disorders according to which these are characterized by an inability to resist to impulses and include disorders such as explosive intermittent disorder, kleptomania, pyromania, pathological gambling and trichotillomania [35]. This comprehension seems pretty far from the different definitions of impulsivity mentioned earlier and the literature on impulse control disorders following TBI is scarce. We have however identified some studies on kleptomania [36] and pathological gambling [37,38,39] that are specific to the TBI population. It should also be noted that Wood and Thomas mention that different forms of post-TBI aggression with different aetiologies (such as impulsive and episodic aggression) are not differentiated in the DSM IV and are considered to be explosive intermittent disorder [9]. The authors add that this does not allow for a distinction of concepts that are not equivalent.

#### 3.1.3. Inhibitory Control

Inhibitory control has been associated to orbitofrontal damage and has been linked to the ability to engage in goal-directed behavior. Patients with damage in this region are abnormally distractible, and have difficulty controlling impulsivity and instinctual behavior [40]. More specifically, the author mentions that patients who have suffered from damage to the orbital prefrontal cortex have abnormalities that seem to be the result of deficits in inhibitory control such as altered emotions and cognitions and emotional and social behavior.

According to Wood, the ability to self-regulate social behavior is undermined by reduced inhibitory control of emotion and behavior and a lack of inhibitory control results in a tendency to act impulsively without thought of consequences and is often associated with a lack of concern for social values [41]. This lack of concern reflects a change of personality following TBI (more specifically to the orbito-frontal cortex) and is often referred to as pseudo-psychopathy or acquired sociopathy. According to the author, these personality changes are usually associated with poor social judgment and short-lived enthusiasm for ill-judged projects, euphoric mood, sometimes accompanied by emotionally labile and erratic behavior, with low tolerance of frustration, leading to irritability and impulsive aggression.

Also, a few other authors have related a lack of inhibitory control to impulsive aggression following TBI. More specifically, Lishman used the concept of inhibitory control to distinguish impulsive aggression from episodic aggression following TBI [42]. According to this author, a lack of inhibitory control can be explained by defective modulatory mechanisms associated with injuries in the prefrontal cortex as opposed to episodic aggression which is of a neurochemical nature. Similarly, Grafman and colleagues theorized that a lack of inhibitory control is the underlying cause of impulsive aggression [43].

The term *inhibitory control* has also been used in relation to emotional regulation. Cattran, Oddy and Wood define emotional regulation as the ability to exercise inhibitory control over how we express and/or direct our emotions in different forms of social interaction [44]. According to these authors, inhibitory control is the mechanism that allows anticipatory reactions that help us judge the consequences of our behavior and a lack of inhibitory control is associated, according to the authors, with emotionally labile and impulsive behavior, often in the form of irritability and poor temper control. In this study, the authors developed a questionnaire to measure emotional regulation after acquired brain injury called the BIRT (Brain Injury Rehabilitation Trust) Regulation of Emotions Questionnaire (BREQ).

#### 3.1.4. Inhibition/Disinhibition

In most of the articles consulted for the following review, it seems that disinhibition is synonymous with impulsivity. As an illustration of this, in Neuropsychological assessment, when searching for disinhibition, the authors invite the reader to see also impulsivity [45]. Similarly, according to Constantinidou, Wertheimer, Tsanadis, Evans and Paul, both impulsivity and disinhibition belong to the same domain of executive functions: Initiation and planning [46]. Also, in a study evaluating conversational abilities in TBI patients, impulsivity and disinhibition constitute one factor representing impulsive or disinhibited conversational behaviors (saying rude or embarrassing things) [47]. More specifically, this factor encompasses speaking too quickly, saying or doing things others might consider rude or embarrassing, allowing people to assume wrong impressions from the conversations, answering without taking time to think about what the other person has said, having trouble using a tone of voice to get the message across, getting “sidetracked” by irrelevant parts of the conversation and losing track of conversations in noisy places.

For Luria, however, disinhibition is the general background on which are superimposed euphoria, impulsiveness and inadequate emotional actions [48]. According to his model of frontally-mediated changes in personality and emotion, disinhibition is one of the two general sets of impairments (the other one being inhibition and torpidity).

For Hanna-Plady, failure to inhibit behavioral responses that are inappropriate to the environmental contingencies or fail to lead to successful goal attainment are frequent after frontal lobe injury [49]. The author adds that this lack of inhibition often presents as behavioral impulsivity.

For Fuster, disinhibition is characterized by distractibility, difficulty in focusing and concentrating and difficulty in inhibiting interference of irrelevant stimuli [40]. Serebro-Sorek, Shakhar and Hoofien use this definition in their study on basic attentional impairments in TBI patients and add that symptoms of disinhibition are accompanied by hyperactivity, impulsive behavior, inappropriate social behavior and unpredictable changes in affect [50]. In this study, the authors measured disinhibition with the Behavioural Assessment Questionnaire (BAQ) in which the disinhibition items assessed the patient’s ability to reasonably plan activities, postpone her/his needs, be calm and not irritated by minor events and his or her ability to resist distractions. This questionnaire was completed by the patient’s neuropsychologist.

As mentioned earlier, the DEX is commonly used questionnaire to assess a variety of executive dysfunctions. As for the impulsivity item, disinhibition is evaluated with a single question. The item that specifically measures disinhibition evaluates if the patient says or does embarrassing things in the presence of others.

Another measure of frontal dysfunction commonly used is the Frontal Systems Behavioural rating scale (FrSBe) [27,51,52,53] and measures three domains of frontal dysfunction: Apathy, disinhibition and dysexecutive symptoms. The disinhibition scale is measured by 15 items evaluating encompassing a wide range of behaviors such as laughing or crying too easily, doing embarrassing things, making sexual comments, swearing, doing things impulsively, being overly silly, acting inappropriately, talking out of turn, not getting along with others, doing risky things, being easily angered or hyperactive, getting into trouble with the law, loss of taste or smell and lacking sensitivity to others.

On a more cognitive level, Rieger and Gauggel use a definition of inhibition in which inhibition is measured as a deliberate and complete suppression of an ongoing motor response [54]. The authors, therefore, target an intentional form of inhibition. The authors also add that this type of inhibition requires one of the most extreme forms of control and is required in many real life situations, where unanticipated changes in the environment suddenly make ongoing actions inappropriate. In other words, an incapacity to suppress an ongoing response might lead to inappropriate behavior in specific situations. According to the authors, in most studies this form of inhibition is measured with the Go/No-Go task. Similarly, Braun, Daigneault and Champagne demonstrated that paradigms designed to elicit commission errors (such as the go/no go paradigm or a paradigm with prestimulus warning) were the most sensitive to distinguish severe chronic TBI patients from controls [55].

#### 3.1.5. Dyscontrol

Lux, in an article exploring the different chronic neuropsychiatric manifestations of TBI, describes behavioral dyscontrol as being a lost or diminished regulation in the behavioral sphere that is characterized by impaired social judgment and difficulty regulating emotional function as it contributes to and integrates with behavioral output [56]. According to the author, agitation is frequent in cases of behavioral dyscontrol, however not all cases of post TBI agitation relies on the same mechanisms. He also encourages clinician to differentiate a difficulty in integrating emotional factors and social judgment from posttraumatic delirium and from episodic dyscontrol or intermittent explosive disorder which, according to him, are all manifestations of behavioral dyscontrol, but have distinct aetiologies.

In a study on emotional change following TBI, the authors base their comprehension of behavioral and emotional dyscontrol [57] on Kinsella, Packer and Olver’s classification of post-TBI difficulties according to which impulsivity, aggression, short-temperedness and self-centeredness are reflections of poor self-monitoring and dyscontrol (behavioral and emotional) [58]. Tate also used that conceptualization in a study in which she opposed loss of emotional control to loss of motivation [59].

In a study on neuropsychological complications following TBI, the authors indicate that major features of behavioral dyscontrol include lability, impulsivity and a tendency to act without regard for consequences and that it may occur in both the acute and chronic stages after TBI and in patients with different levels of severity (mild, moderate and severe brain injury) [60]. Behavioral dyscontrol can be measured with validated behavioral dyscontrol scales [61].

#### 3.1.6. Regulation Deficits

Deficits in regulation can either be emotional, behavioral or cognitive. Authors have demonstrated that different types of regulation most likely rely on different types of mechanisms [44].

Firstly, unlike disinhibition and impulsivity which, according to some authors, belongs to the initiation/planning domain of executive functions, self-regulation belongs to the regulation/effective performance domain [46]. Similarly, Callahan defines self-regulation as self-awareness and self-monitoring [62]. In a study on the awareness of deficits in patients who have suffered from TBI [63], the authors used the Self-Regulation Skills Interview [64] which is a semi-structured interview measuring emergent awareness, anticipatory awareness, strategy generation, strategy-use and strategy effectiveness.

Also on self-regulation, Hanna-Paddy mentions that self-regulatory dysfunction can take the form of difficulty in comprehending the emotional consequences of behavior, behavioral disinhibition, or self-awareness involving the inability to be aware of one’s own mental state [49]. According to the author, self-regulatory dysfunction can also have an effect on the appreciation of humor, the ability to take another individual’s perspective, and the use of appropriate judgment in social behavior.

In a study aiming to compare the patient’s and the caregiver’s assessment of the frequency of behavioral problems after TBI [65], the authors used the Head Injury Behaviour Rating Scale (both the self-rating scale and the relative version) [66]. This questionnaire is composed of two subscales composed of 10 items each: The Emotional Regulation subscale and the Behavioral Regulation subscale. The emotional regulation subscale measures impatience, being depressed, anger, anxiety, irritability, being argumentative, being overly sensitive, sudden mood changes, frequent complaining and aggression. The behavioral regulation subscale measures impulsivity, difficulty in becoming interested in things, lack of motivation, poor decision making, childishness, poor insight, being overly dependent, lack of control over social behavior, lack of initiative and irresponsibility.

Finally, in a study aiming to dissociate impulsivity and risk-taking on a behavioral level, the authors suggest that impaired behavioral regulation encompasses both impulsivity and risk-taking behaviors [18].

#### 3.1.7. Risky Behavior

In a study assessing different characteristics in veterans with a history of TBI and substance use disorders [67], the authors use Zuckerman’s definition of risky behaviors as the tendency to engage in behaviors that have the potential to be harmful or dangerous but which may be perceived by the individual engaging in the behavior as an opportunity to obtain a positive outcome, such as short-term pleasure [68]. Zuckerman adds that thrill/adventure seeking, experience seeking, disinhibition, and susceptibility to boredom are considered to be important components of risky behavior.

### 3.2. The General Concepts Associated with Post-TBI Impulsivity

In the last paragraphs, we first listed different terms that seemed related to the construct of impulsivity following TBI. We then reviewed several studies in order to define each of these words. We first noticed that different definitions are used for the same label and that different labels can also be used for a similar definition. Also, we have observed that in some cases the definitions were either weak or circular, thus not helping the reader clearly understand what each of this concepts refers to. In order to make some sense out of these definitions, we proceeded in the opposite way by initially ignoring the label and looking for common themes/concepts that stood out from the definitions and seeing what they were associated to. This approach allowed us to identify four distinct general concepts related to post-TBI impulsivity. The first concept found in the literature refers to the notion of acting without thinking or preconsideration and has been associated with labels of impulsivity, impulsiveness and disinhibition. This concept is usually conceived as multidimensional. The second concept relates to the inability to control, inhibit or suppress ongoing motor, behavioral or emotional responses as well as interference from distractions. The preservation of general knowledge of the correct behaviors is also found among the definitions. This concept has been associated with various labels such as impulse control, inhibitory control, dyscontrol, disinhibition and emotional regulation. As opposed to the inability to inhibit which seems at face value to refer to a unitary process or to be unidimensional, the third concept refers explicitly to a group of executive functions responsible for the regulation of behaviors in accordance with environmental constraints. Among these functions, we found self-awareness, self-monitoring, anticipation, planning and implementation of action. This broader concept has been associated with labels of regulation deficits, behavioral regulation, inhibitory control, dyscontrol and emotional regulation. The last concept, sometimes called risky behaviors, relates to the narrower notion of sensation seeking. To simplify the labelling, we named these four concepts as impulsivity, deficit of inhibition, deficit of behavior regulation and sensation seeking. Table 1 shows terms, overt manifestations and measures that have been associated with these post-TBI impulsivity concepts.

**Table 1:** General concepts related to post-TBI impulsivity.

Concept	Names Associated with Concept	Overt Manifestations	Multi Dimensional	Measures Specific to the Concept
Acting without thinking or preconsideration (impulsivity)	Impulsivity	Acting too quickly, quick decision	Yes	Questionnaires: BIS-11, UPPS
		Acting without evidence of preconsideration		
	Impulsiveness	Present orientation		Structured interview: BDSI^1^
		Inability to remain focused on a task		Rating scale: IRS^2^
	Disinhibition	Acting on strong impulses under affective condition		Direct observation
		Sudden movements		Tasks: Discounting delay
		Discounting delay		
Inability to control, inhibit or suppress ongoing motor, behavioral or emotional responses or interference from distractions (deficit of inhibition)	Impulse control	Rigid behaviors, perseveration, stimulus-boundedness, hyperactivity	No	Tasks: SART, GNG^3^
	Inhibitory control	Inappropriate social behaviors, lack of concern for social values, poor social judgement, lack of sensitivity to others, speaking or acting too quickly, being rude or embarrassing, inability to use environmental cues, risky behaviors		Questionnaire: BIRT-BREQ, HIBRS-ER^4^ subscale, BAQ
	Dyscontrol	Poor control in the expression of emotions, emotional labile, unpredictable changes of mood, low tolerance of frustration or delay of need gratification, impatience, irritability and aggression, overly sensitive, argumentative, depressed, anxiousness		
	Disinhibition	Distracted or lost in conversation		Rating scales: FrSBe
	Emotional regulation	Lack of planning		
Deficits of executive functions responsible for the regulation of behaviors in accordance with environmental constraints (deficit of behavior regulation)	Regulation deficits	Lack of self-awareness of one’s strengths and weaknesses	Yes	Questionnaire: HIBRS-BR^5^ subscale
	Behavioral regulation	Inability to understand the impact of actions on others, to take another individual’s perspective		Structured interview: SRSI^6^
	Inhibitory control			The 6 elements task (BADS^7^)
	Dyscontrol	Lack of goal-directed behaviors, self-monitoring, anticipation, strategies, implementation of activity		Qualitative variables of specific tasks
	Emotional regulation			
Tendency to seek sensation (sensation seeking)	Sensation seeking	Tendency to engage in behaviors that have potential to be harmful or dangerous	No	Questionnaire: UPPS, Zuckerman
	Risky behavior	Thrill/adventure seeking, new experience seeking, susceptibility to boredom		

Notes: ^1^ Behavioral Dysexecutive Syndrome Inventory; ^2^ Impulsivity Rating Scale; ^3^ Go/ No Go; ^4^ Head Injury Behaviour Rating Scale, emotional regulation; ^5^ Head Injury Behaviour Rating Scale, behavioral regulation; ^6^ Self-regulation Skills Interview; ^7^ Behavioral Assessment of the Dysexecutive Syndrome.

### 3.3. Distinctions and Similarities

Based on the preceding four concepts related to post-TBI impulsivity, we analyzed the main distinctions and similarities between these constructs.

Firstly, the theoretical backgrounds of these concepts belong to different fields in psychology. Both impulsivity and sensation seeking are derived from the literature on personality and may reflect a tendency or a way of being as opposed to the deficit of inhibition or behavior regulation associated with a post-TBI syndrome which are derived from the neuropsychological literature and are a result of function loss after TBI.

Closely related to the distinction between a tendency *versus* a loss of function, the second distinction between these concepts relates to the nature of the consequences of each concept. Both impulsivity and sensation-seeking can have both positive and negative consequences. For example, a person who makes quick decisions can be seen as being spontaneous and a person who scores highly on the sensation-seeking dimension can be seen as being open to new experiences. This is less the case for deficits of inhibition and behavior regulation which are mainly associated with negative outcome. A few exceptions to that statement can be found occasionally when family members report a personality improvement in their post-TBI relative [69].

Another important distinction between these concepts relates to the level of conceptualization of impulsive phenomena. The definitions of impulsivity and sensation seeking are mainly concerned with behavioral tendencies whereas the ones of deficit of inhibition and behavior regulation try to capture the cognitive processes underlying these behaviors. This is an important distinction because it organizes the way one can see the relationships between these constructs. For example, impulsivity can be seen as the result of deficit of inhibition or behavior regulation, as well as other processes such as biological processes related to the temperament. Conversely, impulsivity can be seen as a necessary consequence of deficit of inhibition or behavior regulation. Indeed, a lack of inhibition or anticipation (a function of behavior regulation) must necessarily result in acting without thinking or preconsideration. This way of conceiving the relationships between constructs can help to resolve some of the overlaps between post-TBI impulsivity-related concepts found in the literature.

Table 2 shows a summary of the important distinctions among the four general concepts listed above.

**Table 2:** Similarities and distinctions between general concepts related to post-TBI impulsivity.

Concept	Origin of Impulsive Behaviors	Level of Conceptualization	Relation to other Concepts	Consequence	Theoretical Background
Impulsivity	Tendency	Behavior	Could be the result of deficits of inhibition or behavior regulation, but other processes as well	Positive and negative	Personality
Deficit of inhibition	Function and/or ability loss	Cognitive Processes	Impulsivity is a necessary consequence of deficit of inhibition. Inhibition is one dimension of executive functioning	Negative with a few exceptions	Neuropsychology
Deficit of behavior regulation	Function and/or ability loss	Cognitive processes	Impulsivity is a necessary consequence of deficits of behavior regulation. Behavior regulation composes a group of executive functions	Negative	Neuropsychology
Sensation seeking	Tendency	Behavior	One dimension of impulsivity	Positive and negative	Personality

### 3.4. Fit with the UPPS Model

As mentioned earlier, the UPPS model is a multidimensional model of impulsivity [7]. In an effort to include the UPPS model, which has been receiving a significant amount of support in the literature and has been used with various populations, in the post-TBI literature we tried to see if any of the general concepts we have identified could be linked to the different dimensions.

Theoretically, considering that the UPPS model is a model of impulsivity, all of the dimensions fit into the first concept which we also summed up as being impulsivity. However, the concept of acting without thinking or preconsideration is closer to the dimension lack of premeditation. 

The concept surrounding deficits of inhibition seems to be linked mainly to the urgency, (lack of) perseverance and (lack of) premeditation dimensions of the model. It appears to us that if the ability to inhibit certain responses or some distractions is diminished, the patient will be less able to resist acting on strong impulses under conditions of affect (urgency) and less capable of remaining focused on a task that may be boring or difficult (lack of perseverance). Of course, a deficit of inhibition can also result in a difficulty in reflecting on the consequences before engaging in an act (lack of premeditation). Lack of planning has in fact been mentioned as being an overt manifestation of this concept.

The concept encompassing a group of executive functions responsible for behavioral regulation seems to be linked mainly to the complex dimension of (lack of) premeditation. It appears logical to us that if the patient has a difficulty in the anticipation of a goal-directed behavior, he/she might have a difficulty in reflecting on consequences before acting.

Finally, sensation-seeking is obviously linked to the UPPS dimension of the same name which refers to the tendency to enjoy activities that are exciting and to the willingness to try new experiences.

In sum, at face value at least, there seem to be some bridges between the UPPS model of impulsivity and the four general concepts that stood out in the literature. Indeed, each post-TBI impulsivity concept is reflected in one or many UPPS dimensions. However, until the cognitive processes underlying each of the UPPS dimensions are better understood, the four general concepts remain relevant to explain post-TBI impulsivity. 

### 3.5. Distinction with Attention

Difficulties related to attention are frequent following TBI [70] and could be similar, in certain cases, to impulsivity. For example, a person could leave a meal he or she was cooking unattended and burn it. It can become difficult to distinguish whether that act can best be explained as being impulsive or an error in attention. Some authors have tried to differentiate these concepts.

Fuster made a distinction between patients who have suffered from lesions to the dorsolateral cortex and patients who have suffered from lesions to the orbitofrontal cortex [40]. He describes that both have attentional deficits in the foreground, but that the latter is better described as being distractible due to an inability to inhibit interference from external stimuli, making these patients labile and unpredictable.

For Luria (1980), the distinction is related to the patient’s level of awareness [71]. According to this author, an act can be considered as being disinhibited when the patient is awake and alert and knows better. In other words, the patient knows what the correct behavior is, but does not use this knowledge [72].

For O’Keeffe and colleagues however, an impulsive error occurs in a context where the outcome is not predictable (*i.e.*, in a random task). In their study aiming to differentiate errors due to a failure of inhibitory control from attentional errors on a sustained attention to response task (SART), the authors hypothesized that impulsive errors (due to a failure of inhibitory control) represent a response inhibition failure and are not the same as errors of sustained attention, the former being observed in random sequences of the SART, the latter being observed when the participant slips into a routine during a predictive sequence [52]. The authors add that an impulsive error can be more easily monitored.

In a study aiming to identify attentional impairments in two subgroups of post-TBI patients, one apathetic, the other disinhibited, the authors demonstrated that the apathetic group, as identified by the Behavioural Assessment Questionnaire, had attenuated response to novel stimuli and faster habituation rates than the disinhibited group [50]. Therefore, this study suggests that there is a link between disinhibition, at least when assessed by the Behavioural Assessment Questionnaire, and attention.

In sum, a clear distinction between attentional impairments and impulsivity does not seem to exist in the current post-TBI literature as it does in the UPPS model where a lack of perseverance includes an attentional component. However, there seems to be a consensus that these are distinct constructs that have an influence on each other.

## 4. Discussion

This review shows that there are many concepts that are closely related to post-TBI impulsivity. However, a consensual definition does not exist for any of them. There are multiple inconsistencies in the definitions associated to impulsivity. Indeed, many authors use different terms as synonyms to describe the same concept and the same term might also describe different concepts. Also, the complexity of each concept differs. For example, for some authors, impulsivity is a complex multidimensional concept that taps into different executive functions such as planning and self-monitoring, and for others, impulsivity is conceptualized as a more narrow concept, the selection of a smaller reinforcer that comes in a shorter delay rather than a delayed larger reinforce, for example. It is therefore important for each author to define what he or she is studying and that is not done systematically. In fact, for the current review, many articles in which the authors have mentioned different concepts associated with impulsivity did not define what exactly they were referring to which makes it difficult for researchers to make associations between studies.

We did however find four common themes and concepts in the definitions of post-TBI impulsivity-related concepts. These general concepts seem to account for most of the definitions linked to impulsivity in the post-TBI literature. We have noticed that there are two theoretical backgrounds that explain impulsivity after traumatic brain injury: The personality literature and the neuropsychological literature. Depending on the angle the author chooses, the measures differ. The former usually calls for questionnaires, either self-report or relative-report, or direct observation, the latter usually calls for neuropsychological tasks. Of course, each of these methods has its advantages and disadvantages. For example, direct observation is costly in terms of time and resources and it is impossible to observe all contexts of the patient’s life. Questionnaires are usually subjective and subjected to memory biases and to difficulty related to one’s introspection, both of which can be altered post-TBI. Performance tasks sometimes lack specificity and are often hard to translate into real-life deficits. Also, each of these theoretical backgrounds has its strengths and weaknesses. The neuropsychological framework is usually more objective and has a better understanding of the underlying mechanisms, but the personality framework has a global vision and takes into account the individual in his environment.

In the current review, we have demonstrated that the UPPS model [7] seems to have a moderately good fit with the four general concepts that we outlined. This shows that, as is the case with other populations, it is a promising model for the conceptualization and understanding of post-TBI impulsivity. It also might be helpful in bridging the gaps between the neuropsychological and the personality frameworks.

It should be noted that in the studies used for the current review, there are major differences when it comes to delays after the injury. Indeed, the delays post-TBI in which the subjects were evaluated vary significantly from study to study, but also, in some cases, in the same study. Some authors evaluate the patient only a few weeks after the accident, while others evaluate the patient years later. Not only is this important because of the evolution of impulsive symptoms over time, but also because of the contingencies in different settings. For example, in a rehabilitation setting, the patient’s behaviors are usually monitored more closely than they are once that same patient goes back to his home environment which leaves less room for impulsive, inappropriate or dangerous behaviors. We invite authors to take this into account when it comes to selecting timeframes post-TBI.

Finally, it is important to note that the current review did not specifically take into account all the various behaviors that might be considered as impulsive by some authors such as sexually inappropriate behaviors, pathological gambling, aggressive or suicidal behaviors.

## 5. Conclusions

There is a multitude of terms, definitions and measures that are used to assess impulsivity and other related terms. However, most of these terms and definitions can be classified under one of four general concepts. The impulsivity and the sensation-seeking concepts seem to be derived from the psychiatric/personality literature and the deficit of inhibition and deficit of behavioral regulation seem to be derived from the neuropsychological literature. Our study supports the use of a multidimensional model of impulsivity such as the UPPS model to better understand post-TBI impulsivity.
